# Consequences of Irregular Tuberculosis Treatment: A Case Report of Destroyed Lung Syndrome

**DOI:** 10.7759/cureus.50888

**Published:** 2023-12-21

**Authors:** Ahsan A Faruqi, Harshad Patel

**Affiliations:** 1 General Medicine, Dr. D. Y. Patil Medical College, Hospital & Research Centre, Pune, IND

**Keywords:** destroyed lung syndrome (dls), anti-tubercular treatment (att), tuberculosis (tb), collapse, pulmonary tuberculosis sequelae, pulmonary tuberculosis, destroyed lung

## Abstract

Destroyed lung syndrome (DLS) refers to the irreversible and complete destruction of lung tissue, often due to chronic or recurrent lung infections. Pulmonary tuberculosis (PTB) is a prominent cause of this condition, particularly prevalent in regions burdened by high PTB rates. This report delineates the case of a 60-year-old Indian male who presented with DLS as a consequence of a history of irregular PTB treatment. The patient complained of a productive cough, hemoptysis, fever, and dyspnea. A comprehensive evaluation confirmed the diagnosis, prompting the re-initiation of antitubercular therapy. This case report highlights the challenges and consequences of irregular PTB therapy leading to severe lung damage, emphasizing the significance of prompt and consistent treatment in preventing such debilitating outcomes.

## Introduction

Tuberculosis (TB) continues to pose a considerable public health challenge on a global scale that contributes significantly to mortality and morbidity, particularly in endemic regions, such as India [1]. The 2023 WHO Global TB Report highlighted a concerning increase in TB cases reported in 2022, which reached 133 cases per 100,000 population, totaling 10.6 million reported cases [2].

Destroyed lung syndrome (DLS) is a condition characterized by irreversible and extensive lung damage, often resulting from chronic or recurrent lung infections, particularly TB. In addition, causes can include aspergillosis, bronchiectasis, multiple lung abscesses, and necrotizing pneumonia. It represents a severe end-stage pulmonary disease characterized by extensive destruction of lung tissue and architecture. This syndrome typically manifests after inadequate or irregular treatment of TB, leading to significant impairment of lung function and presenting a considerable challenge in management [3].

This case pertains to a 60-year-old Indian male who presented with symptoms of productive cough, fever, and exertional dyspnea associated with loss of appetite and weight reduction. The patient had a history of pulmonary tuberculosis (PTB) from a decade ago, during which he underwent four months of antitubercular therapy but failed to complete the full course. Following a comprehensive assessment involving clinical, radiological, and laboratory examinations, a diagnosis of DLS resulting from incomplete PTB treatment was made. Subsequently, he was re-initiated on anti-tubercular treatment.

## Case presentation

A 60-year-old Indian male from a low socioeconomic background presented with complaints of productive cough, hemoptysis, fever, and dyspnea. The patient’s dyspnea initially was grade 1 as per the modified Medical Research Council Dyspnea Scale and progressed to grade 4 within four months. The cough was intermittent and persisted for nearly two months, associated with whitish expectoration occasionally streaked with blood. Moreover, the patient reported an insidious evening rise in temperature for three weeks without associated chills or rigors, subsiding upon taking acetaminophen. In addition, he reported a significant loss of appetite and a weight reduction of nine kilograms over four months. A decade ago, he was diagnosed with drug-sensitive, microbiologically confirmed PTB. Although antitubercular therapy was commenced, the patient discontinued the medication after four months, when his symptoms subsided, and he was later listed as lost-to-follow-up. The patient did not reveal any past substance abuse or smoking.

Upon examination, the patient appeared cachexic, with a BMI of 14.04, was afebrile, with a pulse rate of 84 beats per minute, blood pressure of 100/70 mmHg, and oxygen saturation of 94% in room air. On inspection and palpation, decreased lung expansion was noted on the left side compared to the right. During auscultation, reduced breath sounds with crepitations were observed on the left side, while normal vesicular sounds were heard on the right side.

**Figure 1:**
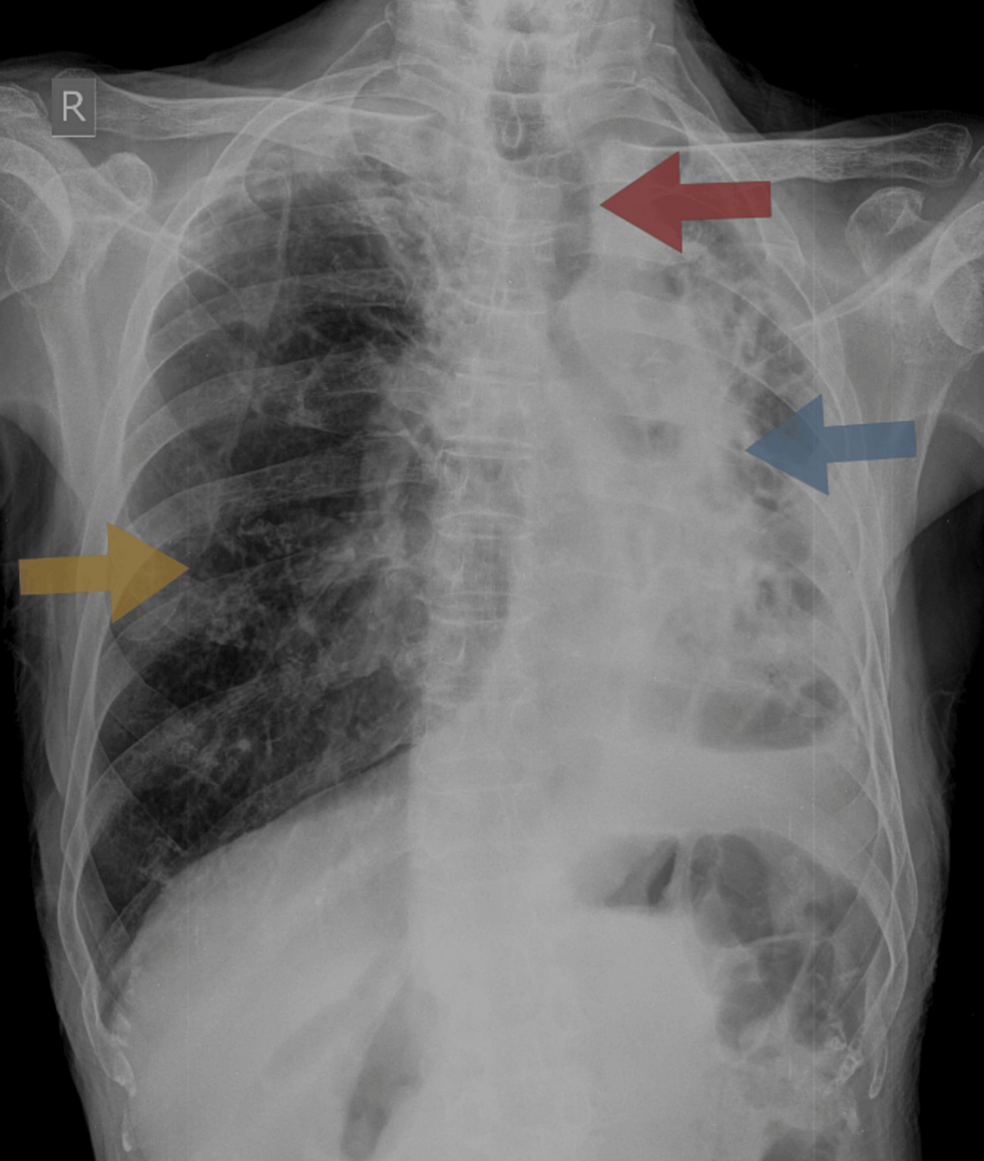
X-ray of the chest shows significant loss of volume in the left lung with distortion of architecture (blue arrow), leading to mediastinal and tracheal shift toward the ipsilateral side (red arrow) with compensatory hyperinflation of the right lung parenchyma (yellow arrow)

**Figure 2:**
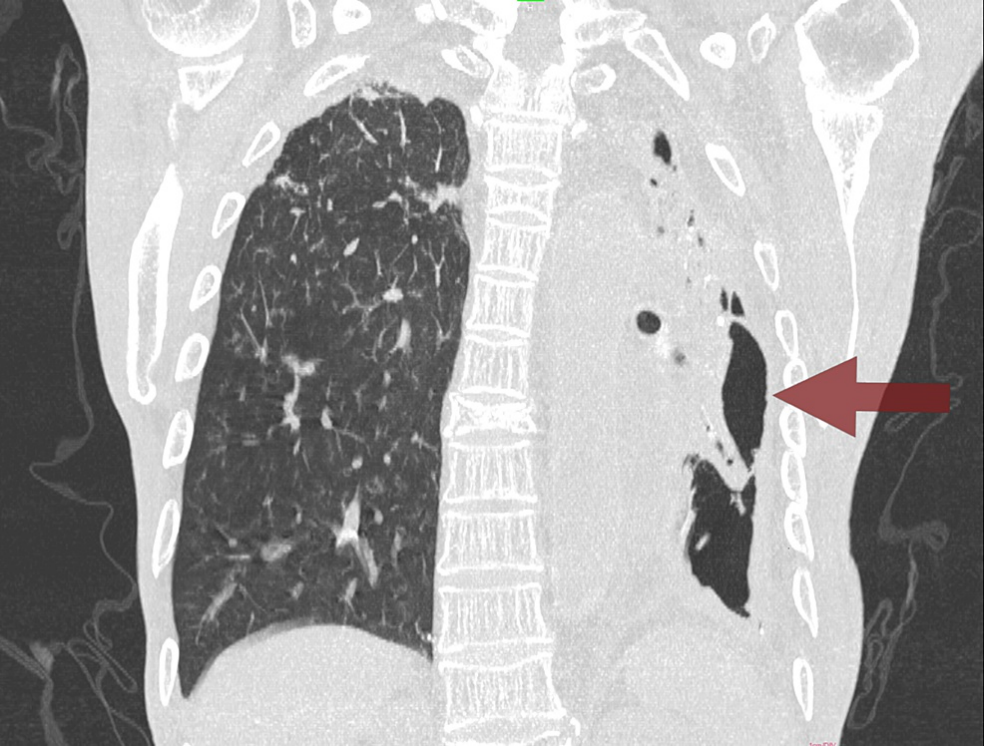
CT chest in the coronal section reveals a significant decrease in the volume of the left lung (red arrow) CT: computed tomography

**Figure 3:**
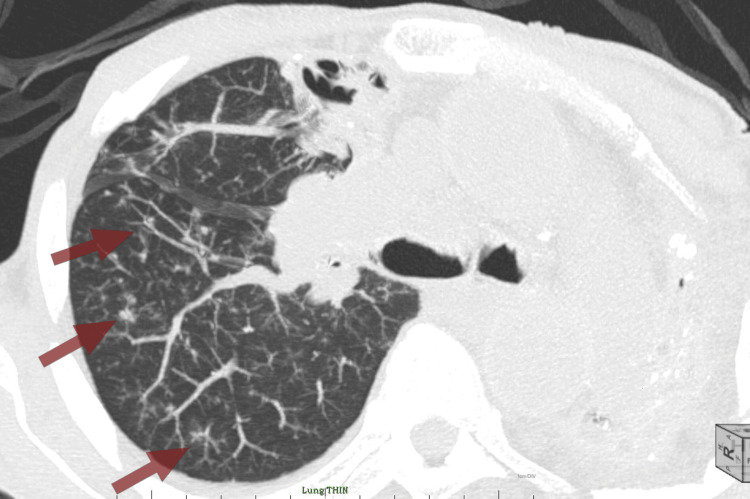
CT chest in the axial section reveals multiple "tree in bud" opacities (red arrows) in the right lung and significant left lung volume loss CT: computed tomography

An initial diagnosis of PTB prompted further investigation. Routine blood tests came back normal, except for a raised erythrocyte sedimentation rate. Microscopic examination and cultures of sputum did not reveal any acid-fast bacilli or fungal hyphae growth. However, through cartridge-based nucleic acid amplification, *Mycobacterium tuberculosis* was detected with sensitivity to rifampicin. Other tests, such as the serum galactomannan, *Mycobacterium* other than tuberculosis panel, routine urine analysis, thyroid profile, and HIV status, all returned normal results (Tables 1, 2). Both the chest X-ray and CT scan displayed complete collapse and consolidation of the left lung, causing the trachea to deviate toward the left. In addition, the right lung exhibited compensatory hyperinflation (Figures 1, 2). Furthermore, centrilobular nodules with a tree-in-bud appearance were evident in the lower regions of the right lung, indicative of active PTB (Figure 3). Pulmonary function tests showed signs of an obstructive disease pattern. The forced expiratory volume in 1 second (FEV1) measured 0.89 L, the forced vital capacity (FVC) was 1.62 L, and the FEV1/FVC ratio was 55%.

**Table 1:** Routine blood investigations SGOT: serum glutamic-oxaloacetic transaminase; SGPT: serum glutamic pyruvic transaminase; ESR: erythrocyte sedimentation rate; HIV: human immunodeficiency virus

Parameters (normal limit)	Report
Hemoglobin (13.2-16.6 gm/dl)	14 gm/dl
Total leucocyte count (4,000-10,000 /µL)	7400 /µL
Platelet count (1,50,000-4,10,000 /µL)	2,03,000 /µL
Serum urea (17–49 mg/dL)	38 mg/dL
Serum creatinine (0.6–1.35 mg/dL)	0.77 mg/dL
Serum bilirubin (0.2–1.2 mg/dL)	1.0 mg/dL
SGOT (8–48 IU/L)	44 IU/L
SGPT (7–55 IU/L)	48 IU/L
ESR (up to 20 mm/hr)	66 mm/hr
Random blood sugar level (up to 140 mg/dl)	103
HIV antibody	Negative
Hepatitis B antibody	Negative
Hepatitis C antibody	Negative

**Table 2:** Additional investigations AFB: acid-fast bacilli; MOTT: *Mycobacterium* other than tuberculosis

Test	Report
Sputum routine microscopy	No acid-fast bacilli or hyphae
Sputum culture	No growth
Cartridge-based nucleic acid amplification	*Mycobacterium tuberculosis* detected without rifampicin resistance
AFB-MOTT blood panel test	Negative
Serum Galactomannan	Negative
Beta-D-Glucan Assay	Negative

After diagnosing PTB with destroyed lung syndrome, the treatment followed the National Tuberculosis Elimination Program's guidelines. This included a four-drug antitubercular treatment with isoniazid, rifampicin, pyrazinamide, isoniazid, and ethambutol [4]. The patient was commenced on nebulization using respules containing a combination of levosalbutamol 1.25 mg and ipratropium bromide 500 mcg thrice daily to ease dyspnea, alongside a nightly dose of 200 mg of acebrophylline. Thorough counseling was provided regarding treatment adherence and the importance of regular follow-ups at the nearest health center. After 14 days, the patient reported an improvement in symptoms and was discharged with instructions to return for a follow-up at the outpatient department.

## Discussion

The bacterium *Mycobacterium tuberculosis* causes TB, a disease that continues to be a significant global health concern [2]. Although it usually affects the lungs, it can also damage the kidneys, spine, brain, and other organs. The symptoms include a persistent cough, fever, gradual weight loss, and nocturnal sweating. It can lead to various complications and long-term sequelae, especially if left untreated or incompletely treated. In pulmonary tuberculosis (PTB), the disease may progress to more severe forms, such as cavitary TB, where the lung tissue is destroyed, increasing the risk of recurrent infections and further spread of the disease. Chronic lung damage, known as fibrosis, may persist, affecting respiratory function even after a successful treatment [3].

DLS refers to extensive damage to the lungs resulting from pulmonary diseases, primarily infections. Although these conditions can be treated, they often leave behind significant complications. TB, despite being curable with chemotherapy, remains a primary cause. In addition, certain severe lung infections treated with antibiotics may also lead to similar consequences. Individuals with "destroyed lungs" can be asymptomatic initially. However, approximately a decade after the initial disease, they may experience various issues, such as progressive difficulty in breathing leading to irreversible respiratory problems, frequent lung infections, and instances of coughing up blood, all of which increase the risk of aspergillosis. Radiologically, "destroyed lungs" show opacities, multiple cavities, or a large cavity damaging both pleural and lung tissue damage, leading to cavities, bronchiectasis, reduced lung volume, and a mediastinal shift toward the affected side [5].

Osarenkhoe et al. reviewed 31 published cases of DLS, revealing that the left lung was the most commonly affected, accounting for 58.1% of cases. In addition, the condition appeared to be more common in males [6]. Another study by Fawibe et al. in Nigeria highlighted high mortality rates due to unilateral lung destruction, emphasizing the importance of early hospital presentation [7].

In 2023, Yadav published a case resembling the current one, involving a 22-year-old male diagnosed with DLS. Both cases share similarities in incomplete TB treatment history, breathing difficulties, affected lung side, ethnicity, sterile culture, and specific findings, such as mediastinal shift, lung destruction, and over-inflation of the opposite lung [8]. However, disparities exist between the current and their case regarding age, the presence of a productive cough, hemoptysis, and a positive cartridge-based nucleic acid amplification report.

The management of DLS lacks formal treatment guidelines. Medical strategies predominantly focus on symptom relief, often using combinations of long-acting beta-2 agonists or long-acting muscarinic antagonists with inhaled corticosteroids to achieve bronchodilation. In addition, pulmonary rehabilitation programs offer tailored exercises and education to enhance lung function and alleviate symptoms. Oxygen therapy is often necessary to improve blood oxygen levels. Vaccinations against influenza and pneumonia are crucial to prevent respiratory infections that can worsen the condition. Regular monitoring is essential to detect any worsening symptoms or complications, such as recurrent infections [9].

Interventions, such as bronchial artery embolization (BAE) might become necessary when hemoptysis fails to resolve following medical therapy or when a patient presents with massive hemoptysis. However, the use of this procedure remains a topic of debate. In a retrospective review of 288 patients with PTB who experienced hemoptysis and underwent BAE, Kim et al. concluded that the risk of rebleeding was notably high, particularly in patients with DLS [10]. On the other hand, Hwang et al., in their review of 93 patients in South Korea, found that BAE was highly effective in promptly controlling bleeding associated with active TB or post-TB sequelae. They further observed that a second BAE could effectively manage even instances of rebleeding [11].

The use of surgical procedures, such as pneumonectomy, for treating severely damaged lungs remains debatable due to the elevated risk of postoperative complications. Li et al. conducted a retrospective study involving 137 patients with DLS who underwent pneumonectomy. They found that strict adherence to surgical indications, thorough preoperative preparation, and precise operative techniques led to favorable long-term outcomes [12].

Preventing DLS involves vigilant monitoring of patients and consistent adherence to antitubercular medication regimens. Regular counseling and support are crucial because of the challenges posed by a high pill burden, lengthy treatment duration, and potential adverse drug reactions. Comprehensive supervision, education, and support throughout the treatment process are crucial to minimize the risk of incomplete treatment and reduce DLS incidence.

## Conclusions

This case report underscores the critical consequences of irregular tuberculosis treatment, leading to the development of DLS in a 60-year-old male patient. The comprehensive evaluation and subsequent diagnosis highlight the severity of lung damage resulting from inadequate therapy, emphasizing the crucial need for consistent, complete, and timely anti-tubercular treatment. Managing DLS remains challenging due to the absence of formal guidelines. Awareness of the significance of treatment adherence and vigilant monitoring is pivotal in averting the debilitating outcomes associated with this condition. Raising awareness among healthcare providers and the community about the implications of incomplete tuberculosis treatment is crucial to preventing the development of DLS and its devastating consequences.
